# Spectral Features of Canthaxanthin in HCP2. A QM/MM Approach

**DOI:** 10.3390/molecules26092441

**Published:** 2021-04-22

**Authors:** Kevin Clark, Natalia B. Pigni, Kithmini Wijesiri, José A. Gascón

**Affiliations:** 1 Department of Chemistry, University of Connecticut, Storrs, CT 06269-3060, USA; kevin.l.clark@uconn.edu (K.C.); natalia.pigni@uconn.edu (N.B.P.); kithmini.wijesiri@uconn.edu (K.W.); 2 Instituto de Ciencia y Tecnología de Alimentos Córdoba (ICYTAC-CONICET), Ciudad Universitaria, Córdoba X5000HUA, Argentina

**Keywords:** HCP2, QM/MM, canthaxanthin

## Abstract

The increased interest in sequencing cyanobacterial genomes has allowed the identification of new homologs to both the N-terminal domain (NTD) and C-terminal domain (CTD) of the Orange Carotenoid Protein (OCP). The N-terminal domain homologs are known as Helical Carotenoid Proteins (HCPs). Although some of these paralogs have been reported to act as singlet oxygen quenchers, their distinct functional roles remain unclear. One of these paralogs (HCP2) exclusively binds canthaxanthin (CAN) and its crystal structure has been recently characterized. Its absorption spectrum is significantly red-shifted, in comparison to the protein in solution, due to a dimerization where the two carotenoids are closely placed, favoring an electronic coupling interaction. Both the crystal and solution spectra are red-shifted by more than 50 nm when compared to canthaxanthin in solution. Using molecular dynamics (MD) and quantum mechanical/molecular mechanical (QM/MM) studies of HCP2, we aim to simulate these shifts as well as obtain insight into the environmental and coupling effects of carotenoid–protein interactions.

## 1. Introduction

Photosynthetic organisms produce a specific type of pigment known as carotenoids. Although carotenoids are highly hydrophobic, their solubility is increased by binding to proteins. Once bound to photosynthetic related proteins, carotenoids can adopt specific orientations, functioning as accessory pigments by absorbing excess photons not captured by other chromophores [1]. In addition, carotenoid-binding proteins play protective roles such as stopping the formation of reactive oxygen species (ROS) by dissipating excess light energy or directly quenching singlet oxygen [2,3]. Many functions and structures of carotenoid-binding proteins require further study and are still being identified to this day [4].

Orange Carotenoid Protein (OCP) is part of an exceptional photoprotective mechanism in cyanobacteria. It has two well-defined domains with a ketocarotenoid spanning both domains. The N-terminal domain (NTD), unique to cyanobacteria, consists of an all α-helix structure and acts as the “effector” domain [5]. Recently, there has been an increased interest in sequencing cyanobacterial genomes, which has resulted in the identification of new OCP families as well as homologs of both domains [6,7,8]. To date, at least nine different NTD homologs have been discovered with diverse functionalities [4]. These homologs are known as Helical Carotenoid Proteins (HCPs). They present similar helical folds to those described for the NTD of OCP and also have the ability to bind carotenoids. Homologs to the C-terminal domain (CTD) of OCP, known as CTDHs, have also been found in almost every genome encoding HCPs [4,7]. Protein evolution studies, combining gene fusion with taxonomic species distribution, have suggested that OCP was likely derived from a domain fusion event between an HCP and a CTDH [5,6,8,9].

In this work, we focus our investigation on HCP2; one of the most widespread subtypes of the HCP family found in cyanobacteria. Recently, it has been isolated from *Tolypothrix* sp. and its crystal structure was reported with a 1.7 Å resolution (PDB: 6MCJ) [9]. HCP2 exclusively binds canthaxanthin (CAN) and is an effective singlet oxygen quencher. However, unlike the active form of OCP, it is unable to bind to the phycobilisome (PBS) to quench excess energy [9]. In solution, HCP2 is found as a monomer, while the crystal structure reveals a dimeric structure with a 5 Å separation between the stacked β1-rings of the two CAN molecules. The UV–visible absorption maximum of the crystal structure of HCP2 is red-shifted by 18 nm when compared to HCP2 in solution (548 nm and 530 nm, respectively), and both are significantly red-shifted (>50 nm) with respect to CAN in solution [4,9]. The shape of the carotenoid absorption bands is directly related to the conjugation of the polyene chain and the conformation of the terminal rings [10,11,12].

Experimental studies of CAN in solution have been performed using different solvents, including benzene, methanol and *n*-hexane [9,13]. The observed absorption maxima vary slightly depending on the solvent. Besides the shift in the absorption maximum, the absorption lineshape of HCP2 in solution is very similar to that of the isolated CAN in solution. This suggests that the carotenoid ending rings have a comparable freedom to rotate inside the protein [14]. In this work, we aim to explain the spectral features observed in experimental studies by means of quantum mechanical/molecular mechanical (QM/MM) and excitonic coupling calculations. Additionally, we use molecular dynamics (MD) simulations in order to reveal the interactions and conformations associated with the UV–vis spectral features of the complex in solution.

## 2. Results and Discussion

### 2.1. QM/MM Calculations of HCP2 Models from the Crystal Structure

The crystal structure of HCP2 (PBD: 6MCJ) was used as the starting point for our QM/MM calculations. The protein complexes were prepared with Maestro (Schrödinger 2019-1), using the entire PDB structure for calculations of the dimer, and the two individual chains separately (A and B) for calculations of the monomeric structures. We performed a local minimization of the carotenoid in the QM region, leaving the protein frozen. On average, this local minimization produced only small changes of the polyene chain in the protein cavity (~0.20 Å).

**Figure 1:**
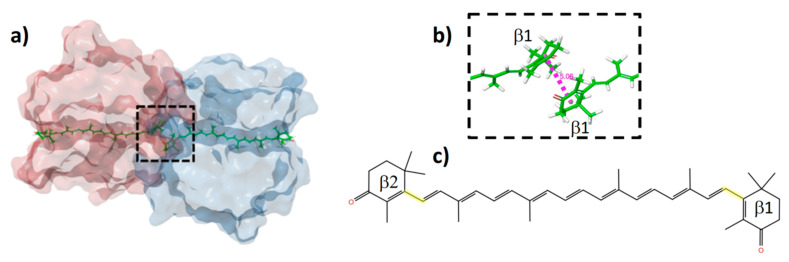
HCP2 dimer model (PDB: 6MCJ). (**a**) Protein surface representation with each monomer colored differently and canthaxanthin (CAN) molecules shown as green sticks. (**b**) Enlarged view of the dashed square in panel (**a**) to highlight the close interaction between the two β1 rings. (**c**) CAN with labeled β1 and β2 rings. Highlighted in yellow are the bonds for which dihedral angles will be discussed throughout this work.

Figure 1a shows the HCP2 model from the crystal structure. The dimer is composed of two monomers, each non-covalently bound to a CAN molecule. The β1 rings of both carotenoids are placed in close proximity (~5 Å) showing a stacked alignment. Due to this interaction, the solvent-exposed area of the carotenoids is reduced when compared to the isolated monomers. It has been demonstrated that the main oligomeric state of HCP2 in solution is as a monomer. Thus, in solution, it is expected that the chromophore experiences an enhanced conformational freedom, in contrast to the dimeric structure where the protein environment constrains the β1 terminal ring rotation [9].

During the system preparation process of the dimer (previous to the QM/MM calculations), we found one particular glutamic acid (Glu94), located in the proximity of β1 ring (<5 Å), which became protonated (neutrally charged) after hydrogen-bonding optimization at pH 7. In contrast, the same procedure applied to each individual monomer assigns a negatively charged state for Glu94. It is already known that the pKa values of ionizable groups of acidic and basic residues can be affected by the protein microenvironment, differing from the normal values observed in water [15]. Interestingly, this particular residue is the closest charged amino acid to the β1 terminal ring in the monomer. As previously discussed, the β1 terminal rings have significant environmental differences between monomeric and dimeric structures.

Post-optimization single-point QM/MM excitation energy calculations were performed on both oligomeric states of HCP2, and the results are shown in Table 1. Note that the monomers extracted from the crystal structure show a 14 nm difference between the calculated λ_max_ values, which is probably explained by the existence of different conformations in β1. The end-ring torsions in β-carotene-like molecules are known to have a marked influence on the electronic properties and excitation energies [16]. In the case of CAN in solution, despite the freedom to rotate, the lowest energy conformations are predominant with torsions around 45°, restricting the conjugation of the system to ~465–480 nm absorption maximum values, depending on the particular solvent [9,13]. Red-shifted states occur for a completely planar conjugated system, *s*-cis (0°) or *s*-trans (180°), but this is prevented in solution by intrinsic steric interactions between the rings and the methyl groups of the isoprenoid chain. In the crystal structure of HCP2, the β2 rings of both monomers are nominally *s*-trans, while the β1 rings show a nominally *s*-cis configuration. After performing a QM optimization of CAN inside the protein environment, both dihedrals maintain the same nominal conformation with dihedrals of ~179° for β2. However, the *s*-cis β1 ring torsions reveal different values for each monomer favoring a larger conjugation for ligand B, which is reflected in a red-shifted absorption maximum (Table 1), in much closer agreement to the experimental absorption λ_max_ (530 nm). Thus, we conclude that the best crystal structure to construct solvated models of the of HCP2 in its natural form is monomer B.

Regarding the experimental 18 nm shift observed between the λ_max_ of the dimer (548 nm) and monomer in solution (530 nm) [9], the results from our calculations on the crystal structures show a calculated shift of 23 nm (comparing the dimer with monomer B), which is in very good agreement with the experimental result.

### 2.2. Distortions, Environmental Influence, and Excitonic Coupling

**Figure 2:**
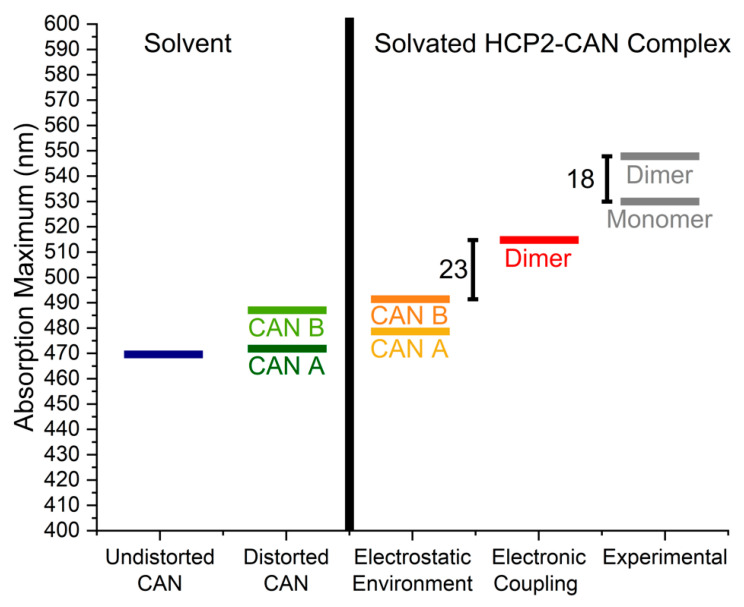
Absorption maxima calculated for canthaxanthin (CAN) after QM optimization in different environments using implicit solvent: CAN alone (undistorted), CAN isolated from the protein (distorted), and CAN inside HCP2 in two different oligomeric states. Experimental values are from reference [9].

There are three effects on the absorption spectrum that were evaluated: (1) the mechanical distortion as a result of change in conformation from solvent to protein, (2) the combined electrostatic and screening effects due to the protein and solvent, and (3) the effect of electronic coupling between the two chromophores. Figure 2 illustrates how these effects progressively affect the excitation energy. While there are no experimental values of the excitation energy of CAN in water, we still considered CAN in water theoretically to be able to see the effects as CAN becomes surrounded by the protein cavity in an aqueous environment. For monomer A, the mechanical distortion exerted by the protein produces a minimal change in absorption. This is in part due to the large out of plane value of β1 (74°) that breaks conjugation. On the other hand, the mechanical distortion of CAN in monomer B produces a substantial red shift due to a more nominally *s*-cis conformation in β1 (37°), which favors conjugation.

The electrostatic environment of the protein plus solvent causes a very small red shift of 3–5 nm in the calculated λ_max_ for both monomers. This observation also applies to other related carotenoid-containing proteins (e.g., OCP, RCP) [17]. It is interesting to note the contrast with other light-driven proteins, such as type I and II rhodopsins, for which the tuning mechanism of the protein chromophores is strongly influenced by the electrostatic environment [18].

The last effect to consider is the strength of excitonic coupling (i.e., the electronic coupling between chromophores). While this is not relevant for the natural function of HCP2 (which occurs as a monomer in solution), it is worth asking what level of energy transfer we would expect between the two CANs in the crystalline state. To determine this, we computed the electronic coupling between the S_0_ → S_2_ excited states of the two CAN ligands in the presence of the protein and solvent environment using the electronic energy transfer methodology [19,20]. This last effect adds a substantial red shift (23 nm from monomer B), which explains the origin of the observed experimental red shift from solution to crystal. Thus, the 18 nm shift reported experimentally for HCP2 is closely recreated by our QM/MM calculations.

Table 2 shows the site energies (i.e., localized excitons), the electronic energy transfer (EET) coupling between the two localized excitons, and the corresponding eigenvalues of the 2 × 2 exciton Hamiltonian. The accuracy of the coupling calculations (0.061 eV) is evident in the close comparison between the eigenvalues and the two lowest excitation energies of the full dimer calculation at the QM/MM level. It is interesting to compare the magnitude of the excitonic coupling (0.061 eV = 492 cm^−1^) with recent calculations by our group on the antenna peridinin chlorophyl *a* protein (PCP) [21]. In PCP, there is a 4:1 peridinin (Per) to Chl *a* ratio. The biological role of PCP is to funnel excitation energy through paths connecting the four peridinins, ending in a localized exciton on Chl *a* [22,23]. Per–Per–Per and Per–Chl couplings range between ~200 cm^−1^ and ~600 cm^−1^. Thus, the CAN–CAN coupling found in the crystal structure of the HCP2 dimer is as large as the largest couplings in PCP.

### 2.3. Molecular Dynamics of HCP2 Monomers

HCP2 in solution is predominantly found as a monomer in a dynamical equilibrium between multiple conformations, in contrast with the crystal structure that reflects spectral properties of a more restrained conformation. We were interested in evaluating the effect of temperature on the computation of the relevant excitation energies. In order to obtain a sampling of representative states of the complex in solution, we performed 1000 ns long classical MD simulations for the individual monomers of HCP2. In our approach, we kept the general parametrization of the OPLS3e force field, applying a local minimization of the carotenoid for each snapshot under a QM/MM treatment. This procedure has been demonstrated to yield very accurate results for OCP and the Red Carotenoid Protein (RCP), within the range of the reported experimental values [17]. In that previous work, we found that the in-place root mean square deviation (RMSD), before and after QM/MM minimization, was, on average, 0.28 Å. Thus, the local QM/MM minimization, while producing very small displacements within the protein cavity, creates accurate structures that are suitable for the evaluation of spectroscopic properties at the QM level.

The MD simulations for both monomers show stable RMSD values for the protein (~1.8 Å) and CAN (~1.3 Å) during the entire trajectories (Figure S1, Supplementary Materials). Besides the predominant hydrophobic contacts, one of the most frequent protein–ligand interactions is a water bridge with Glu94 (Figures S2 and S3, Supplementary Materials). Similar contacts have been described for RCP, where the carotenoid interacts mostly through hydrophobic contacts between residues of the cavity and the isoprenoid chain, while both rings show an increased solvent exposure when compared to the OCP cavity [17].

From the resulting MD trajectories, 51 snapshots evenly distributed along the simulation time (every 20 ns) were extracted for each monomer. After deleting the solvent and ions, the structures were locally optimized using QSite, including the carotenoid in the QM part, and freezing the entire protein. Single points were run on each optimized snapshot in the presence of implicit solvent to obtain excitation energies. The results are shown in Table 3. The MD simulations allow both monomers to reach more similar average λ_max_ values between them, thus decreasing the shift observed in the crystal structure. However, the absolute absorption maxima calculated show a ~50 nm discrepancy with respect to the experimental value reported for HCP2 in solution. We were puzzled by the fact that this same protocol and same level of theory produced very accurate results in OCP and RCP [17]. Thus, we explored what aspects in the modeling of HCP2 could be deficient.

**Figure 3:**
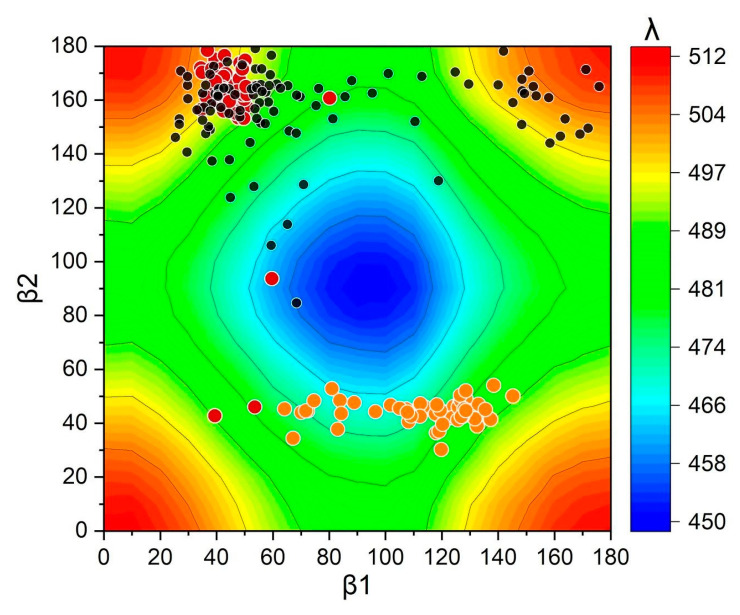
End-ring dihedrals distribution of the optimized snapshots from MD simulations for: OCP^O^ (orange circles), RCP (red circles) [17], and HCP2 monomers (black circles). The underlying contour plot shows the calculated absorption maxima values for a full scan of β1/β2 dihedrals of CAN in vacuum.

As described before, the torsions of the end-terminal rings are among the main factors affecting the conjugation of a carotenoid, which are reflected on its spectral properties [12,16]. Being a homolog of the NTD of OCP, HCP2 is structurally similar to RCP. In solution, the terminal rings of the carotenoids bound to these two proteins are exposed to solvent. However, as we show below, this exposure to solvent does not necessarily result in enhanced conformational freedom for the β1 ring to rotate. Figure 3 shows the conformational surface explored by the end-ring dihedrals of the optimized snapshots for OCP^O^, RCP and HCP2 monomers. The conformations are plotted over an underlying contour graph showing the calculated absorption maxima for a full scan of CAN dihedrals in vacuum (see Methods section for details). The torsions explored by OCP^O^ and RCP are much more restricted (at least in one of the angles) than those observed for HCP2. Particularly, the environment of RCP influences the selection of a subset of end-ring torsions, mostly restricted to a nominal *s*-cis β1 (~45°) combined with a nominal *s*-trans β2 (~160°). This predominant configuration leads to red-shifted absorption maxima in RCP, in agreement with experiments. In contrast, although the conformational surface explored by CAN in HCP2 is restricted to absolute values over ~90° for β2 (nominal *s*-trans), the values are widely distributed within that range. In addition, the variability is even higher for β1, which explores values covering almost the whole range of torsions from 20° to 180°. Only in a small number of snapshots do β1-torsions in HCP2 assume values over ~160°, which combined with similar values of β2-torsions, allows higher absorption maxima. Thus, differently than in our previously reported work, in which we matched the experimental values of RCP and OCP through a similar computational approach [17], here we found a discrepancy between the HCP2-calculated values and those reported from experimental measurements.

Besides the homology between RCP and HCP2, the two crystal structures differ in the relative orientation of the monomers in the dimeric structure. Specifically, in HCP2 the orientation of monomers is such that both CAN molecules are positioned head-to-head, while in RCP (PDB code: 4XB4), both CANs are located in a perpendicular orientation without showing any close contacts between them. In addition, RCP and HCP2 monomeric structures present a different set of missing residues, which are positioned to potentially interact with the β1 ring and may be affecting the conformational freedom in a different manner in both proteins. Therefore, even though we are carrying out MD simulations, it is possible that our starting structure of HCP2 does not fully evolve into its most stable conformation in solution.

Within the context of end-rings torsions analyses in these proteins, we also have included data from an MD simulation of CAN in water to compare the behavior of CAN alone in solution [17]. The distribution of β1 and β2 dihedrals of 1000 snapshots of CAN in water is plotted in Figure S4 (Supplementary Materials) over the same graph shown in Figure 3. As expected, the values for CAN in solution show a wider distribution when compared to CAN inside OCP, RCP, or HCP2. However, besides the expected unlimited freedom to rotate of CAN in solution, there is a marked trend to select values between 60° and 80°. This causes the prevalence of blue-shifted absorption maxima when compared to CAN in any of these proteins. Despite the difficulties in accurately reproducing HCP2 experimental values, our computational approach demonstrates the ability to achieve simulations reflecting the general trends and the influence of the protein environment on the carotenoid spectral features.

## 3. Materials and Methods

### 3.1. Model Preparation

The crystal structure of HCP2 (PDB: 6MCJ) was processed using Maestro (Schrödinger Release 2019-1: Maestro, Schrödinger, LLC, New York, NY, USA). For the monomer calculations of HCP2, each alternative chain was taken from the dimeric structure. Water, pentaethylene glycol, and iodide ions were deleted before using the protein preparation module, which was used for assigning bond orders and adding hydrogens. Optimization of hydrogen bond assignment was performed with default parameters (pH 7.0) and a restrained minimization was applied for hydrogens only.

### 3.2. Structure Optimizations and QM/MM Calculations

QM/MM calculations were run on the processed structures. CAN was defined as the QM region while the rest of the protein was treated at the MM level using the OPLS3e force field [24]. A local optimization was done on CAN to correct the bond length alternation (BLA) of the carotenoid, which is overestimated by the force field. This optimization was performed using QSite (Schrödinger Release 2019-1: Maestro, Schrödinger, LLC, New York, NY, USA) at the DFT B3LYP/LACVP* level with CAN in the QM region and with the protein frozen. Excited state energies were then calculated from the optimized structures using the Tamm–Dancoff approximation (TDA) at the CAM-B3LYP/LACVP* level. This particular combination of functionals for optimization and single point calculations have produced accurate results by us [17,25] and others before [26]. Solvent effects were treated using the Poisson–Boltzmann (PB) implicit solvent in QSite.

For the dihedrals scan of CAN in vacuum, a local geometry optimization was performed at the DFT B3LYP/LACVP* level using Jaguar (Schrödinger Release 2019-3: Maestro, Schrödinger, LLC, New York, NY, USA). A relaxed scan was performed on the optimized structure, fixing one end-ring dihedral and changing the other dihedral from 0° to 180°, sequentially. A single point energy calculation was performed on the configurations of all scan coordinates. Excited state energies for each pair of β1-dihedral and β2-dihedral were calculated applying TDA at the CAM–B3LYP/LACVP* level. The absorption maxima obtained from the scan were red-shifted by a constant value of 25 nm so that the range of absorptions in OCP and RCP lie roughly on top of the green/blue regions and yellow/red regions, respectively.

### 3.3. Electronic Coupling Calculations

Electronic energy transfer (EET) calculations were implemented with the Gaussian 16 program using TDA at the CAM–B3LYP/6-31g(d) level. EET was used to compute coupling constants between the two carotenoids of the HCP2 dimer. Each CAN molecule was defined as a fragment for the EET analysis. To perform these calculations in the presence of the HCP2 protein complex, partial atomic charges from the protein as well as “solvent” charges from the Poisson–Boltzmann Qsite calculation were added as the background charge distribution.

### 3.4. Molecular Dynamics Simulations

The prepared HCP2 monomers were solvated in an orthorhombic box (10 Å buffer size) using the TIP3P water model [27]. The relative orientation of the box with respect to the protein was optimized to produce a minimum number of waters. After this volume minimization procedure, the resulting number of water molecules was ~5800. Na^+^ ions were added to neutralize the negative charge of the system. All minimizations and simulations used the OPLS3e force field for the protein and CAN. MD simulations were run for solvated systems on Nvidia GPU hardware with Desmond (Schrödinger Release 2019-4: Maestro, Schrödinger, LLC, New York, NY, USA). Prior to MD production, the default relaxation protocol was performed as detailed in our previous publication [17]. The total time for each MD simulation was 1000 ns using the NPT ensemble (300 K, 1 atm). Snapshots were extracted every 20 ns and sent to QSite for QM/MM calculations. RMSD and protein–ligand interactions analyses are described in the Supplementary Materials.

## 4. Conclusions

Using the recently reported dimeric crystal structure of the cyanobacterial HCP2 carotenoid-binding protein, we have applied a computational approach, including QM/MM calculations and MD simulations, to study the electronic and molecular properties behind the spectral features of HCP2. Through QM/MM calculations we were able to reproduce the experimental shift observed between the HCP2 crystal structure (dimer) and the protein in solution (monomer). We have analyzed the influence of different environments on the carotenoid spectral features, demonstrating the progressive effects caused by changing from a solvent to a protein–solvent environment, and the effects of the electronic coupling of the two chromophores in the dimer.

The combination of MD and QM/MM methodologies used here has previously demonstrated the ability to reproduce the experimental absorption maxima of reference proteins (OCP and RCP). Despite the limitations in reproducing HCP2 experimental values, our computational approach shows the ability to simulate the general spectral trends of the studied system, as well as the influence of protein environment on the carotenoid spectral features.

## Figures and Tables

**Table 1:** Quantum mechanical/molecular mechanical (QM/MM) calculations on optimized structure of HCP2. The β1 and β2 dihedral angles correspond to the QM/MM minimized structure.

Model	QM/MM λ_max_ (nm)	β1 Dihedrals	β2 Dihedrals	Experimental λ_max_ (nm)
Dimer	515			548 [9]
Monomer Ligand A	478	74.0°	179.3°	530 [9]
Monomer Ligand B	492	37.2°	178.3°	
Shift (Dimer–Monomer)	23 *			18
CAN (in *n*-hexane)	465	40.7°	143.3°	465 [13]
CAN (in water)	470	41.4°	140.3°	N/A

* Reported shift corresponds to the difference between the dimer and the monomer with highest λ_max_.

**Table 2:** Comparison of QM/MM and EET calculations of absorption maxima of HCP2.

Model	Site Energy λ_max_ nm (eV)	EET Coupling (eV)	Eigenvalues (nm)	QM/MM λ_max_ (nm)	Exp. λ_max_ (nm)
Monomer A	486 (2.551)	0.061	481	484	548
Monomer B	511 (2.427)		514	515	

**Table 3:** QM/MM calculations on optimized structures from MD of HCP2 monomers. Results are expressed as the mean of 51 snapshots. One-sample *t* test was used to obtain the confidence intervals.

Model	λ_max_ (nm)	Confidence Interval (95%)		SD	Experimental λ_max_ (nm)
Monomer Lig. A	481.9	478.3	485.5	12.7	530
Monomer Lig. B	477.0	472.4	481.5	16.1	

## Data Availability

The data presented in this study are available on request from the corresponding author.

## Supplementary Materials

The following are available online. Figure S1: RMSD HCP2 Monomers MD. (A) Ligand A. (B) Ligand B, Figure S2: Protein–ligand interactions in HCP2 Monomer–Ligand A, Figure S3: Protein–ligand interactions in HCP2 Monomer–Ligand B, Figure S4: CAN β1/β2 dihedrals scan over calculated absorption maxima.
