# Supplementary material for: Spectral Features of Canthaxanthin in HCP2. A QM/MM Approach
Source: Molecules. 2021 Apr 22;26(9):2441. doi: 10.3390/molecules26092441 (PMC8122715; doi:10.3390/molecules26092441)
Supplement: Supplementary file 1 [file molecules-26-02441-s001.zip › molecules-1182749-supplementary.pdf]

Supporting Information

Spectral Features of Canthaxanthin in HCP2. A QM/MM Approach

Kevin Clark[1], Natalia B. Pigni[1,2], Kithmini Wijesiri[1] and José A. Gascón[1,*]

[1] Department of Chemistry, University of Connecticut, Storrs, Connecticut 06269-3060, USA; kevin.l.clark@uconn.edu; natalia.pigni@uconn.edu; kithmini.wijesiri@uconn.edu

[2] Instituto de Ciencia y Tecnología de Alimentos Córdoba (ICYTAC-CONICET), Ciudad Universitaria, Córdoba X5000HUA, Argentina; npigni@unc.edu.ar

* Correspondence: jose.gascon@uconn.edu

Molecular Dynamics Analysis

1. *RMSD (Root Mean Square Deviation)*

RMSD values were calculated with respect to the starting snapshot of the MD production. Protein RMSD data shown on the left axis of the figures below is referred to the Cα atoms. Ligand RMSD is on the right axis of the graphs shown below, indicating the fluctuation of the carotenoid with respect to the protein. The protein-ligand complex was first aligned on the protein backbone of the reference and then the RMSD of the ligand heavy atoms was measured.

A

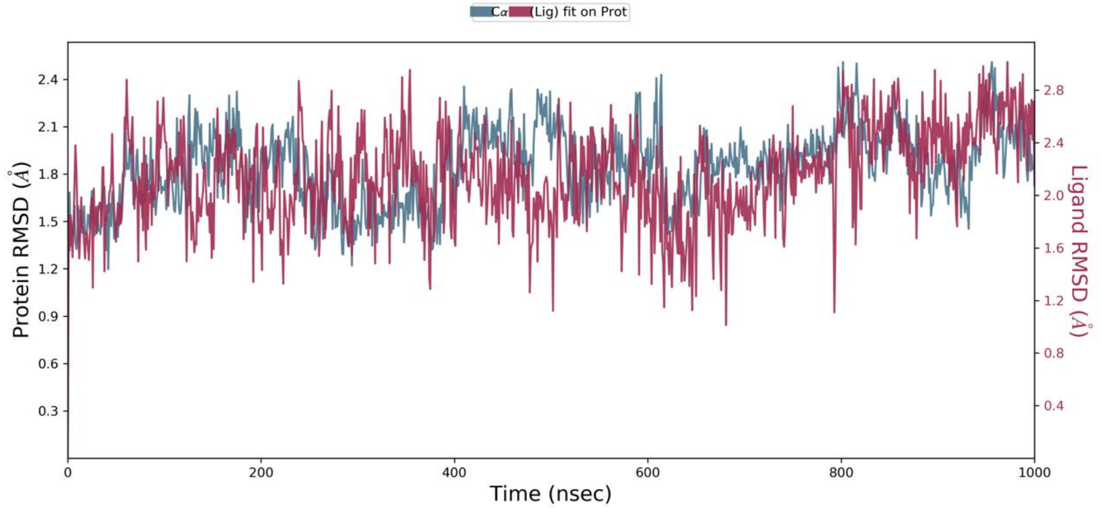

B

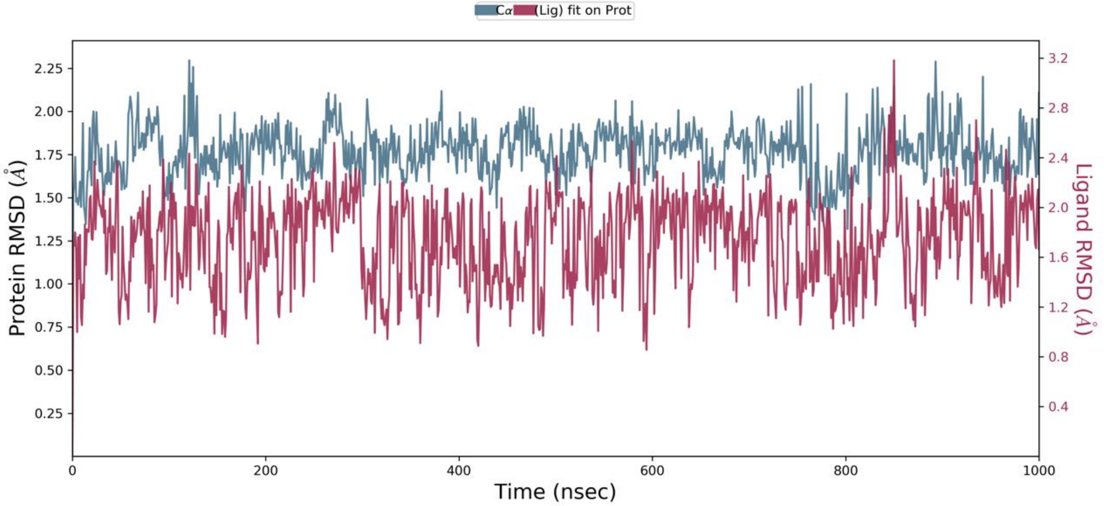

FIGURE S1. RMSD HCP2 Monomers MD. (A) Ligand A. (B) Ligand B

2. *Protein-Ligand Interactions*

Protein-ligand interactions (or 'contacts') are categorized into different types. The interactions observed in our simulations correspond to Hydrogen Bonds, Hydrophobic and Water Bridges. We describe below the criteria for each type of contact.

A representation of the main contacts (>30%) along each MD trajectory is depicted in the upper part of Fig. S2 and S3. The atoms highlighted in gray are the ones exposed to solvent. The stacked bar charts are normalized over the course of the trajectory: the vertical axis indicates the frequency of the interaction along the simulation, a value of 0.7 suggests that 70% of the simulation time the specific interaction is maintained.

- Hydrogen Bonds (green bars in the charts shown below): the geometric criteria for protein-ligand H-bond is: distance of 2.5 Å between the donor and acceptor atoms (D—H···A); a donor angle of ≥120° between the donor-hydrogen-acceptor atoms (D—H···A); and an acceptor angle of ≥90° between the hydrogen-acceptor-bonded_atom atoms (H···A—X).
- Hydrophobic contacts (purple bars in the charts below): Generally, these types of interactions involve a hydrophobic amino acid and an aromatic or aliphatic group on the ligand, but in this analysis, it is extended to include p-Cation interactions. The geometric criterion for hydrophobic interactions is: p-Cation — Aromatic and charged groups within 4.5 Å; p-p — Two aromatic groups stacked face-to-face or face-to-edge; Other — A non-specific hydrophobic sidechain within 3.6 Å of a ligand's aromatic or aliphatic carbons.
- Water Bridges (blue bars in the charts below): are hydrogen-bonded protein-ligand interactions mediated by a water molecule. The geometric criteria for a protein-water or water-ligand H-bond are: a distance of 2.8 Å between the donor and acceptor atoms (D—H···A); a donor angle of ≥110° between the donor-hydrogen-acceptor atoms (D—H···A); and an acceptor angle of ≥90° between the hydrogen-acceptor-bonded_atom atoms (H···A—X).

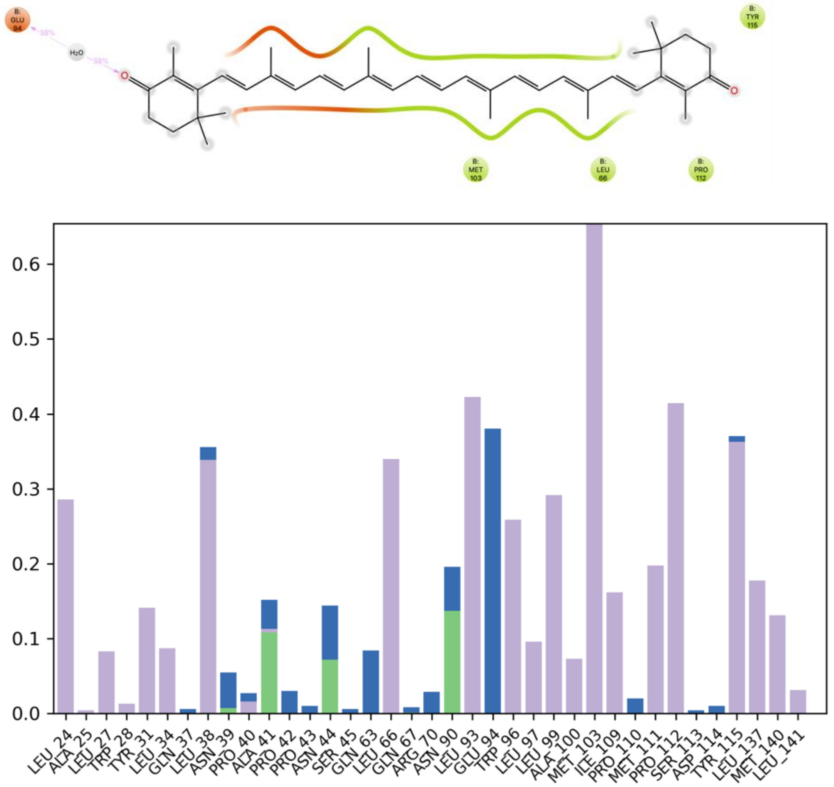

FIGURE S2. Protein-Ligand interactions in HCP2 Monomer - Ligand A.

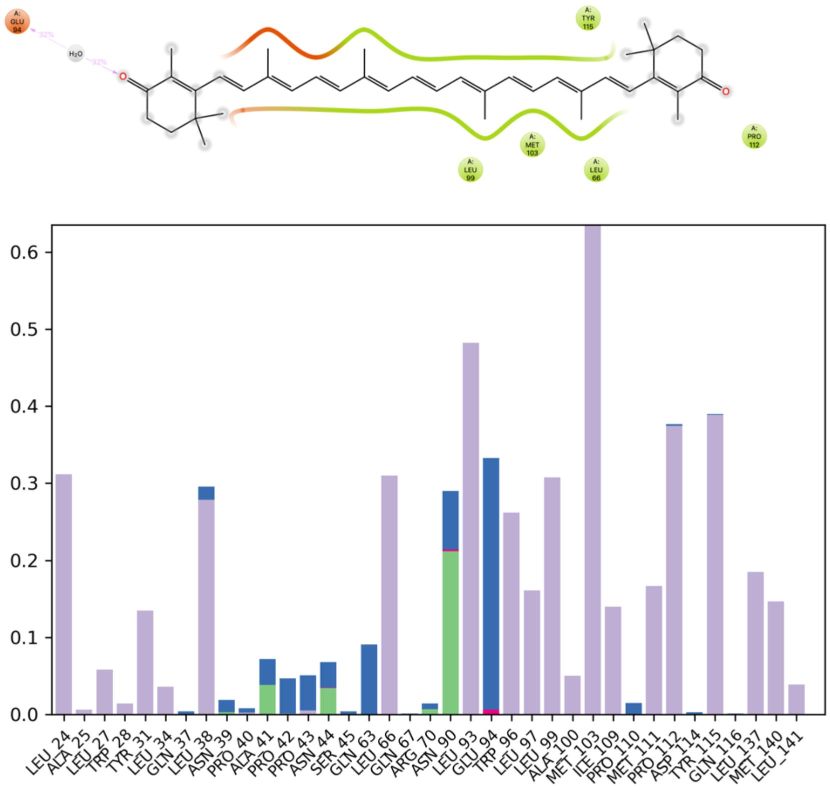

FIGURE S3. Protein-Ligand interactions in HCP2 Monomer - Ligand B

β1/β2 Dihedrals Scan of CAN in Different Environments

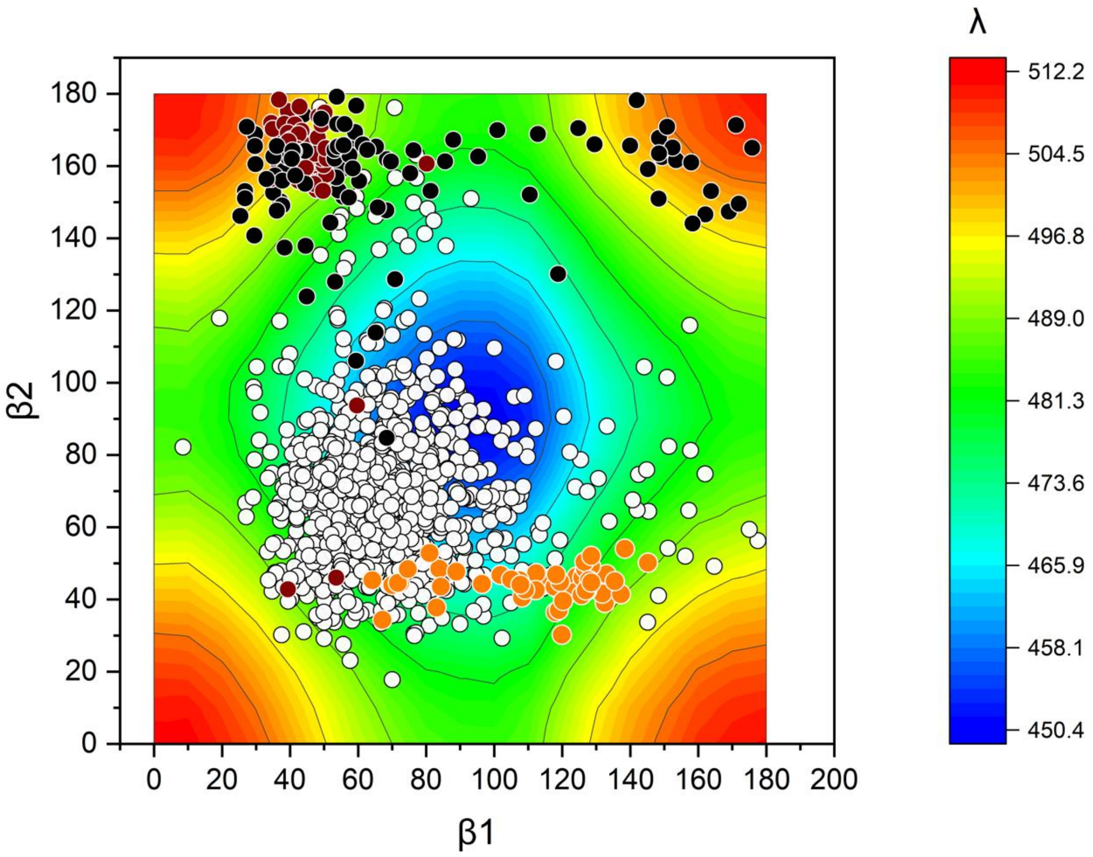

FIGURE S4. CAN β1/β2 dihedrals scan over calculated absorption maxima. End-ring dihedrals distribution of the optimized snapshots from MD simulations for: OCP^O (orange circles), RCP (red circles), HCP2 monomers (black circles), and CAN in water (white circles). The underlying contour plot shows the calculated absorption maxima values for a full scan of β1/β2 dihedrals of CAN in vacuum.
